# Geographic differentiation of ectoparasitic flatworms in the pelagic zone of Lake Tanganyika, Africa

**DOI:** 10.1186/s12983-026-00602-9

**Published:** 2026-05-11

**Authors:** Nikol Kmentová, Kelly J. M. Thys, Christoph Hahn, Jiří Vorel, Lutz Bachmann, Stephan Koblmüller, Maarten Van Steenberge, Auguste Chocha Manda, Lawrence Makasa, Théophile Mulimbwa N’Sibula, Pascal Masilya Mulungula, Maarten P. M. Vanhove

**Affiliations:** 1 Centre for Environmental Sciences, Research Group Zoology: Biodiversity & Toxicology, Hasselt University, Hasselt, Belgium; 2 OD Natural Environment, Freshwater Biology, Royal Belgian Institute of Natural Sciences, Brussels, Belgium; 3 Institute of Biology, University of Graz, Graz, Austria; 4 CESNET z.s.p.o., Prague, Czech Republic; 5 Natural History Museum, University of Oslo, Oslo, Norway; 6 Biology Department, Royal Museum for Central Africa, Tervuren, Belgium; 7 OD Taxonomy and Phylogeny, Royal Belgian Institute of Natural Sciences, Brussels, Belgium; 8 Unité de Recherche en Biodiversité Et Exploitation Durable Des Zones Humides (BEZHU), Faculté Des Sciences Agronomiques, Université de Lubumbashi, Lubumbashi, Democratic Republic of the Congo; 9 Lake Tanganyika Research Unit, Department of Fisheries, Ministry of Fisheries and Livestock, P. O. Box 420055, Mpulungu, Zambia; 10 Département de Biologie, Centre de Recherche en Hydrobiologie, Uvira, Democratic Republic of Congo; 11 Unité d’Enseignement Et de Recherche en Hydrobiologie Appliquée (UERHA), Département de Biologie-Chimie, Institut Supérieur Pédagogique de Bukavu (ISP), Bukavu, Democratic Republic of the Congo

**Keywords:** Population connectivity, Monopisthocotyla, Dorosomatidae, Migration, Habitat preference, PoolSeq

## Abstract

Monopisthocotylan parasites have been proposed as tags for studying host population structure due to their direct life cycles and short generation times. However, their effectiveness in reflecting host population connectivity remains under scrutiny. The poorly-understood connectivity of fisheries stocks in Lake Tanganyika, the second deepest lake in the world with a permanently stratified, relatively species-poor and well-delimited pelagic zone, serves as a case-study to test utility of parasites for fish stock identification. This study investigates the population structure of two *Kapentagyrus* species parasitizing dorosomatid fish in Lake Tanganyika, by analyzing variation in mitochondrial protein-coding genes across a geographic gradient. The study comprised 12 population genomic datasets from the central and southern subbasins of the lake sampled within the same week to account for host migration. We observed differences in geographic population structure of the two parasite species, with restricted gene flow in *Kapentagyrus limnotrissae* infecting a single dorosomatid species and smaller hosts preferring the littoral zone. Conversely, *K. tanganicanus* exhibited no geographical structure, reflecting its broader host range and preference for larger hosts preferring the pelagic zone. The results also highlight that other mitochondrial genes such as those from the dehydrogenase family or *atp6* provide higher resolution for population genetic studies in these parasites than the frequently used *cox1*. The study also compared two sequencing strategies—individual versus pooled sequencing (PoolSeq)—for assessing population structure of monopisthocotylan parasites, and found that PoolSeq yielded similar results with lower demands on individual DNA quantity and sequencing costs. This work supports the use of host-specific, directly transmitted parasites as ecosystem tags and provides valuable insights into the role of host ecology and parasite life-history traits in shaping population dynamics.

**Supplementary Information:**

The online version contains supplementary material available at 10.1186/s12983-026-00602-9.

## Introduction

Lake Tanganyika, Africa, is the second deepest lake in the world. It has a permanently stratified, relatively species-poor and well-delimited pelagic zone. The homogeneity and lack of physical barriers in pelagic ecosystems are believed to promote large distribution ranges and genetic connectivity of organisms [1]. Two endemic dorosomatid clupeiform fish species, Lake Tanganyika sardine (*Limnothrissa miodon* Boulenger, 1906) and Lake Tanganyika sprat (*Stolothrissa tanganicae* Regan, 1917), are common pelagic species and account for approximately 65% of the annual fisheries production of the lake [2, 3]. Given the decline in fisheries production in the lake over the last decades [4–6], accurate stock identification and the establishment of proper management strategies are crucial for sustainable fisheries. In general, the implementation of different data types and approaches in order to correctly identify fish stock dynamics has been recommended [7, 8], also more specifically for the fish stocks of the African Great Lakes [9–11].

Previous studies indicated lake-wide unrestricted migration for both dorosomatid species without apparent spawning ground fidelity [12–14]. However, for *L. miodon,* recent population genomic studies revealed frequency-dependent variation of chromosomal rearrangements across the lake [15]. Such differentiation of fish populations in the absence of significant migration barriers may be driven by selection at adaptive loci [16]. Another recent study indicated that *L. miodon* reproduced year-round in the littoral zone, whereas the reproduction of *S. tanganicae* was seasonal. Furthermore, females of *L. miodon* showed a preference for the littoral, rather than the pelagic zone [17].

Genetic population structure of parasites can provide relevant information for the identification of fish stocks (reviewed in Catalano et al*.* [18]). This has, for example, been shown for discrete stocks of horse mackerel (*Trachurus mediterraneus*) in the Mediterranean Sea [19] or local structuring during prolonged time periods driven by restricted reproductive migration of yellowtail amberjack, *Seriola lalandi* Valenciennes, 1833, along the Chilean coastline [20]. An approach integrating fish and parasite data was also used in stock identification of Pacific sardine, *Sardinops sagax* (Jenyns, 1842) [21], a pelagic commercial species in the southeastern Pacific Ocean [22] or in dorosomatids in Lake Tanganyika [23].

### Microevolutionary dynamics render parasites potential tags for their hosts

The large population sizes of obligate parasites combined with their often short generation times, high fertility, short hatching time, fast sexual maturity and high nucleotide substitution rates facilitate rapid adaptive responses to a changing environment at a microevolutionary level [24–26]. However, obligate parasites depend on their hosts, usually have a limited dispersal capacity and their populations are typically overdispersed with a disproportionate number of parasites concentrated on a small subset of hosts, while most hosts harbour few or no parasite individuals [27]. Accordingly, they may be highly sensitive to changes affecting their hosts [28] and may, therefore, show a fine-grained genetic signature of such events. If true, the genetic structure of parasite populations can serve as a magnifying glass [28] for tracking genetic differentiation of the host. Fisheries pressure, for example, can cause local extinction or reduce genetic variability in fish parasites, with more profound effects seen in directly transmitted and obligate parasite taxa due to their generally overdispersed populations [29, 30]. This has been shown, for example, for the larvae of the nematode *Anisakis simplex* (Rudolphi, 1809) infecting Atlantic herring, *Clupea harengus* Linneaus, 1758 [31, 32].

Population structure and connectivity of parasites infecting pelagic fish species can provide insights into the hosts’ biogeographical connectivity [33], which is often better studied in littoral ecosystems [34]. Yet, studies combining population level genetic differentiation of wildlife parasites with spatio-temporal patterns of their hosts are so far restricted to a few model parasite taxa such as species of *Plasmodium* (Welch, 1897) [35], *Schistosoma* spp. [36], *A. simplex* (s. s.) [32] or plant pathogens such as anther smut fungus *Microbotryum violaceum* Deml & Oberwinkler, 1982 [37]. Despite their proven potential as tags for the history of their hosts [38], monopisthocotylans (parasitic flatworms previously referred as part of Monogenea but see Vanhove et al. [39]) are scarcely considered in this context. This is unfortunate, as their one-host life cycle limits the number of confounding factors involved in their microevolutionary dynamics as compared to other parasites with more complex life cycles.

### Monopisthocotylans as biomarkers in the pelagic zone of Lake Tanganyika

**Fig. 1:**
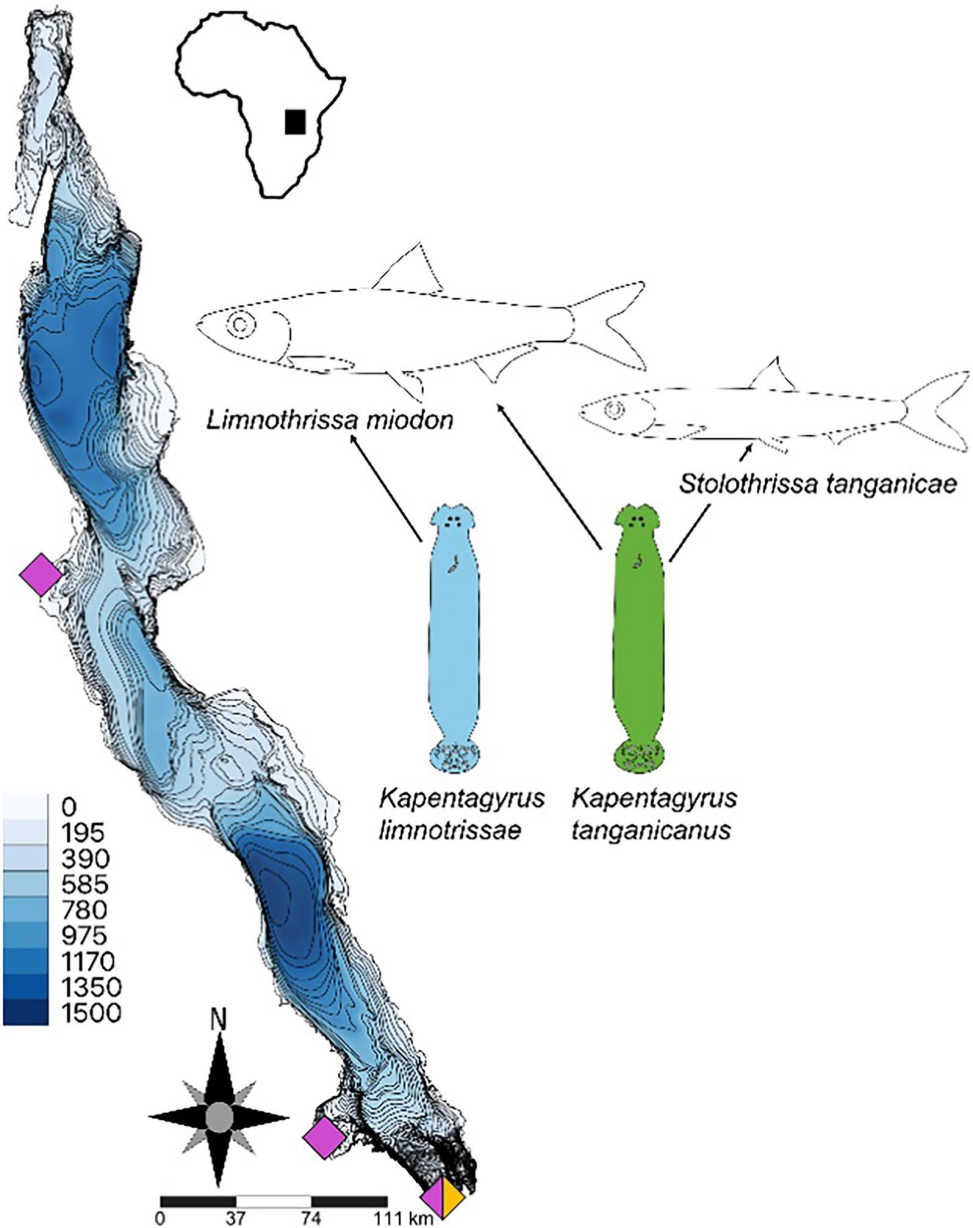
The study system, including host ranges of respective parasite species and sampling localities in Lake Tanganyika. Bathymetry is indicated by color shading (depth in meters), with darker blue representing greater depths. Pink and yellow diamonds represent localities sampled in April 2018 and September 2018, respectively

The two Lake Tanganyika dorosomatid species Lake Tanganyika sardine *L. miodon* and Lake Tanganyika sprat *S. tanganicae* are infected by two monopisthocotylan parasites, both belonging to Dactylogyridae: *Kapentagyrus limnotrissae* (Paperna, 1973) that is host-specific to *L. miodon* and *K. tanganicanus* Kmentová, Vanhove & Gelnar, 2018 that infects both dorosomatid hosts. In dactylogyrid monopisthocotylans, transmission is assumed to occur via outcrossing between hermaphroditic individuals, with eggs being released and hatching in the external environment, and producing free-swimming miracidia that subsequently infect new fish hosts [40]. Population genetic studies targeting a part of the mitochondrial cytochrome *c* oxidase subunit I gene (*cox1*) suggested geographical panmixia of these flatworms [41]. This result corresponded with the lack of geographical structure reported for *S. tanganicae* [12] but contrasted with more recently suggested restricted migration of *L. miodon* [15]. Even so, host lifestyle changes over ontogenetic development and interspecific interactions between these two parasite species structure their spatio-temporal occurrence [23]. Given the limitations of the previously used *cox1* molecular marker and the recently suggested restricted migration of *L. miodon*, we studied the population structure of the parasites *K. limnotrissae* and *K. tanganicanus* across two sub-basins of the lake (Fig. 1) using complete mitochondrial genomes, and tested a Pooled Sequencing (PoolSeq) strategy as an alternative approach to evaluate intraspecific diversity across the mitogenome of monopisthocotylan parasites sampled under field conditions [42–44]. Given that the spatial structure of these two parasites mirrors the contrasting dispersal patterns of their host species’ life stages, we hypothesize that *K. limnotrissae* displays more pronounced geographic genetic structuring than *K. tanganicanus* [23]. The restriction of *K. limnotrissae* to the littoral-associated life stage *of L. miodon* is expected to limit parasite dispersal, a pattern that we should be able to detect using high-resolution mitogenome-level population analyses in contrast to single marker-based reported panmixia [41].

## Materials and methods

### Sampling

Specimens of the dorosomatid fish species Lake Tanganyika sardine (*L. miodon*) and Lake Tanganyika sprat (*S. tanganicae*), both endemic to the lake, were obtained in April 2018 from the Central (Kalemie) and Southern subbasins (Mpulungu and Nsumbu Bay) of Lake Tanganyika, respectively (see Fig. 1 and Table 1). Fish specimens were purchased dead from local fishermen at the landing sites immediately after the overnight catch. The fish specimens were identified to species level in situ following Coulter [45] and stored in absolute ethanol until further examination.

**Table 1:** List of samples with designation of ID, number of individuals being pooled per sample (=pool population size), number of host specimens parasites collected from per pool, sampling locality at Lake Tanganyika with date, subbasin, and number of indexed paired-end reads per pool

Pool ID	# of pooled parasites/collected from # of host specimen	Locality	Subbasin	Date	Host species	# of indexed paired-end reads
*Kapentagyrus tanganicanus* (two host species—infecting *Limnothrissa miodon* and *Stolothrissa tanganicae*)						
A	80/16	Mpulungu	South	12.04.2018	*L. miodon*	42,198,686
B	85/19	Mpulungu	South	12.04.2018	*L. miodon*	757,340,539
C	90/1	Mpulungu	South	19.04.2108	*L. miodon*	770,428,790
D	60/3	Mpulungu	South	19.04.2108	*L. miodon*	487,953,813
E	90/7	Kalemie	Central	12.04.2018	*L. miodon*	699,862,290
F	60/5	Kalemie	Central	12.04.2018	*L. miodon*	417,010,920
G	90/3	Nsumbu Bay	South	12.–19.04.2018	*L. miodon*	707,678,804
I	56/21	Mpulungu	South	12.–19.04.2018	*S. tanganicae*	495,771,135
J	59/19	Kalemie	Central	12.04.2018	*S. tanganicae*	473,418,148
*Kapentagyrus limnotrissae* (host-specific to *Limnothrissa miodon*)						
K	59/17	Mpulungu	South	12.–19.04.2018	*L. miodon*	437,182,681
L	58/2	Mpulungu	South	Sept. 2018	*L. miodon*	497,932,835
M	59/8	Kalemie	Central	12.04.2018	*L. miodon*	566,034,754

Considering potential long-distance migration of fish hosts [12, 46] and to avoid sampling the same population twice, hosts were collected within a maximal timespan of one week in April 2018 (rainy season). To evaluate the seasonal variation in genetic diversity, parasite individuals of *K. limnotrissae* collected in Mpulungu in September 2018 (dry season) were also included. Information on infection parameters of the studied parasite species and sampled populations across Lake Tanganyika including the samples used in this study is summarised in Kmentová et al*.* [23].

Individual parasites were extracted from the gills and transferred to a Petri dish with distilled water. Prior to downstream pooling, the species identity of each monopisthocotylan individual was verified under a DM500 microscope, at magnification 400× using diagnostic features in the attachment organ based on Kmentová et al. [47]. Parasite morphological vouchers containing haptoral structures or the male copulatory organ (sclerotised structures used for morphological species identification, see Kmentová et al*.* [41] for more details) of the parasite specimens included in the pools were mounted on slides in Hoyer’s medium and deposited in the collection of the Research Group Zoology: Biodiversity & Toxicology at Hasselt University (Diepenbeek, Belgium) under accession numbers HU.XX.1.01-HU.XX.4.50, HU.XXI.1.01-HU.XXI.4.50 and HU.XXII.1.01-HU.XXII.2.50.

### DNA extraction, library preparation and mitochondrial genome assembly

Large scale applications of next generation sequencing (NGS) methods on wild populations of monopisthocotylan parasites and other small sized organisms are challenging due to their low quantity of genomic DNA. This issue is further exacerbated in parasites given their limited lifespan after removal from the host or after the host’s death which leads to rapid DNA degradation. Accordingly, population studies on such parasites imply high costs and high risk of failure. Pooled Sequencing (PoolSeq), however, offers to overcome these challenges. It has been shown as a reliable approach to produce population-level genomic datasets of various organisms [48–50] and to evaluate the level of intraspecific nucleotide diversity across the mitogenome of organisms preserved under field conditions [42–44]. Accordingly, prior to extraction of total genomic DNA, individuals of *K. limnotrissae* and *K. tanganicanus* were pooled following Esch et al*.* [51] as either meta- (conspecific parasites of one host species on several host individuals per locality) or infrapopulations (parasites of one species from one host individual per locality). Accounting for PoolSeq being a less powerful method compared to individual-based studies to get information on haplotype frequencies and suffering from possible unequal contribution of pooled individuals, we followed recommendations from Schlötterer et al*.* [49] regarding sequencing coverage (see details below) and to include a minimum number of 50 pooled individuals. Further details can be found in Table 1.

The extraction of genomic DNA from pooled parasite individuals as described in Kmentová et al*.* [43] was followed by library preparation (TruSeq Nano DNA Library Prep, Illumina, no size selection) and sequencing on an Illumina NovaSeq6000 platform (short-insert paired-end sequencing, 2 × 150 bp configuration). Library preparation and sequencing were outsourced to Macrogen Europe with an intended coverage of 2.5×/individual in the pool (A) and 20×/individual in the following pools (B–M) (expected genome size 100 Mb based on Konczal et al*.* [52]) (see Table 1). A sequencing coverage of 20× was targeted following the recommendations of Schlötterer et al*.* [49], a review on the use of the PoolSeq approach. Pool A and pool B, comprising the same number of individuals from the same metapopulation, were sequenced at different coverage depths to assess the impact of sequencing coverage on the results.

To enable comparison between previously reported population genetic structure inferred from individual-based sequencing and the PoolSeq-based approach, variation across multiple mitogenomic regions was analyzed and compared with published results based on the *cox1* region [41]. Prior to the assembly of mitochondrial genomes (mitogenomes), the raw reads were trimmed in Trimmomatic v0.39 [53] using a sliding windows option (window size 3 and quality threshold 26), cutting 5 bases from the start of each read and applying a minimal read length of 100 bp. The mitogenome for each pool/population of *Kapentagyrus* sp. was assembled using part of the c*ox1* sequence of *K. tanganicanus* (GenBank accession number MK598266) and *K. limnotrissae* (GenBank accession number MK598117), respectively, as seed in NOVOPlasty v4.3.1 [54] with a k-mer length of 31, read length of 130 and insert size of 390. In case of non-circularised assemblies, overlapping regions were aligned and trimmed in Geneious Prime 2022.1.1. Mitogenomes were annotated using the MITOS web server (code: echinoderm and flatworm mitochondrial) [55] combined with the tRNAscan-SE [56] and RNAfold [57] web servers to confirm the tRNAcoding regions. Annotation was verified via visualisation of open reading frames and alignment with available mitogenomes of closely related monopisthocotylan monogeneans in Geneious Prime 2022.1.1. The presence and boundaries of repeat regions were investigated with Tandem Repeats Finder [58]. Raw Illumina reads were submitted to SRA (accession numbers: SRR22937639-50) under BioProject accession PRJNA916857.

### Population-level data processing and analyses

Trimmed reads of each pool of mitochondrial reads were mapped back to the assembled consensus mitogenome sequence of the respective pool using default settings in BWA-MEM v0.7.17 [59] followed by removal of PCR duplicates in SAMBLASTER v0.1.24 [60]. Sequence variation across mitogenomes in each population of *Kapentagyrus* sp. was evaluated using a sliding window analysis in PoPoolation v1.2.2 [61] (window size of 300 bp and step size of 10 bp, minimum coverage 4, minimum count 2) excluding AT rich and non-coding regions present between the *nad5* and the *trnG* tRNA genes (see Table S1). Mapped read pools were subsampled to a uniform coverage/individual in the pooled sample to 5× and 10× in PoPoolation v1.2.2 [61]. The number of polymorphic sites, nucleotide diversity (Tajima’s π) and Tajima’s D (used in previous studies dealing with PoolSeq data (see Dennenmoser et al*.* [62] and Kurland et al*.* [63]) were calculated for each pooled population and coverage/individual in PoPoolation v1.2.2 to characterize patterns of genetic diversity and to test for signals of past population changes (Tajima’s D).

Comparison with the previously published *cox1* sequences enabled assessment of the potential of the chosen approach. To evaluate the performance of the PoolSeq approach for *Kapentagyrus* spp. populations, values of Tajima’s π and Tajima’s D for parts of the *cox1* gene (415 bp) were contrasted with individual-based data of *K. tanganicanus* from the same population using a similar number of individuals (GenBank accession numbers MK598125-323 published in Kmentová et al*.* [41]).

Population structure was evaluated between the respective parasite pools across the entire mitogenome as well as for each protein coding gene (PCG) separately (window size of 300 bp and a step size of 10 bp, minimum coverage 5/individual, minimum number of the SNP variant of 2). Population differentiation was inferred by FSTs and Fisher’s exact test in PoPoolation v2 [61]. PCAs were conducted on SNPs shared by all populations and applying either a minimum frequency of the rare SNP variant of 0.01 or 0.05, respectively, using the adegenet R package [64]. The results of all statistical analyses were visualised in RStudio [65], using the ggplot2 [66], ggpubr [67], ggtext [68], ggfittext [69], mdthemes [70], reshape2 [71], factoextra [67], dplyr [72] and tidyverse [73] R packages.

## Results

Genomic high-throughput sequencing of nine populations of *K. tanganicanus* and three populations of *K. limnotrissae* on the Illumina NovaSeq6000 platform yielded sufficient numbers of indexed paired-end reads to assemble mitochondrial genomes with high average sequencing coverage exceeding 7500 (somewhat less in pool A, reflecting the intentionally lower number of reads) (see Table 1 and Fig. S1 in Additional file 1). Complete mitochondrial genomes were 13,323 bp (21.5% GC) and 13,723 bp (21.2% GC) long in *K. limnotrissae* and *K. tanganicanus*, respectively, consistent across different pools, and comprised 12 (all canonical protein coding genes (PCGs) except *atp8*) intron free protein coding genes, 22 tRNA genes and two genes coding for the l2S and 16S subunits of the mitochondrial rRNA (Fig. S2 in Additional file 1).

There were two non-coding regions. An AT-rich non-coding region was located between the *cox2* and *rrnS* genes in *K. limnotrissae* (32 bp) and *K. tanganicanus* (28 bp). The second non-coding region was located between the *nad5* and *trnG* genes, and consisted of an AT-rich segment of 157 bp in *K. limnotrissae* and of 247 bp in *K. tanganicanus*. In *K. tanganicanus*, this was followed by repeated sequences of 125 bp in reverse and forward orientations, with only a forward sequence being present in *K. limnotrissae*. In the mitogenome of *K. tanganicanus*, there was a palindrome sequence of 175 bp between the two repeated sequences of 125 bp mentioned above. Truncated stop codons TA and T and variation in start and stop codons were detected in several PCGs in both species (for more details see Table S1 in Additional file 1).

### Intraspecific variation and population structure

**Fig. 2:**
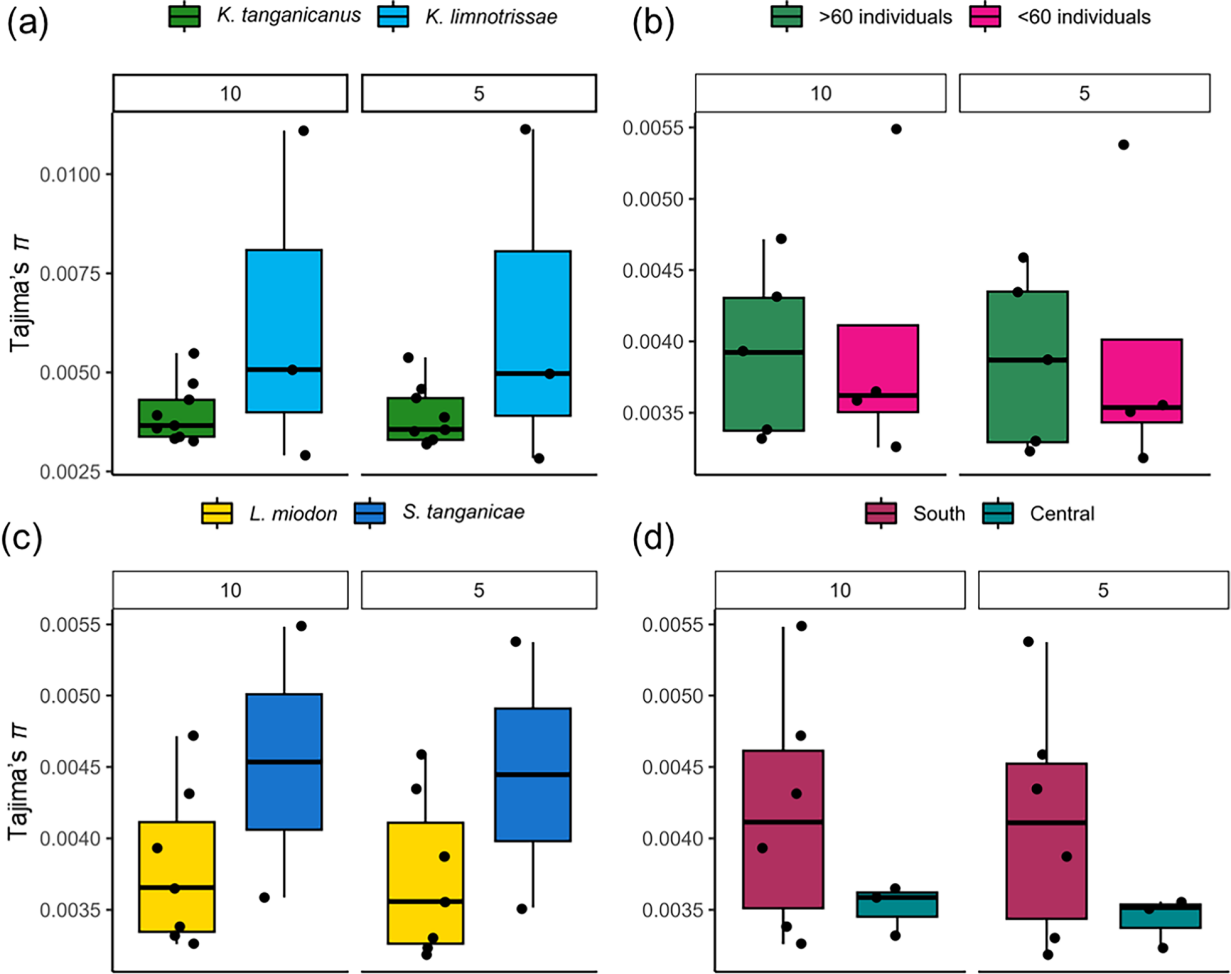
Summary of the nucleotide diversity (Tajima’s π) across all PCGs divided into subsampled coverage/individual. Nucleotide diversity (Tajima’s π) across all PCGs of between species of *Kapentagyrus* (**a**) and in populations of *K. tanganicae* in relation to the pool size (**b**), host-species identity (**c**), and subbasin origin (**d**)

The nucleotide diversity across all PCGs measured by Tajima’s π was slightly higher in *K. limnotrissae* compared to *K. tanganicanus* (see Table S2, Fig. 2a). The nucleotide diversity did not show any correlation with neither the subsampled coverage/individual (Fig. 2) nor the number of pooled individuals (Fig. 2b). In populations of *K. tanganicanus*, the nucleotide diversity appeared to be slightly higher in metapopulations infecting *S. tanganicae* compared to *L. miodon* (Fig. 2c) and in the southern populations compared to the central populations of *K. tanganicanus* (see Table S2, Fig. 2d).

**Fig. 3:**
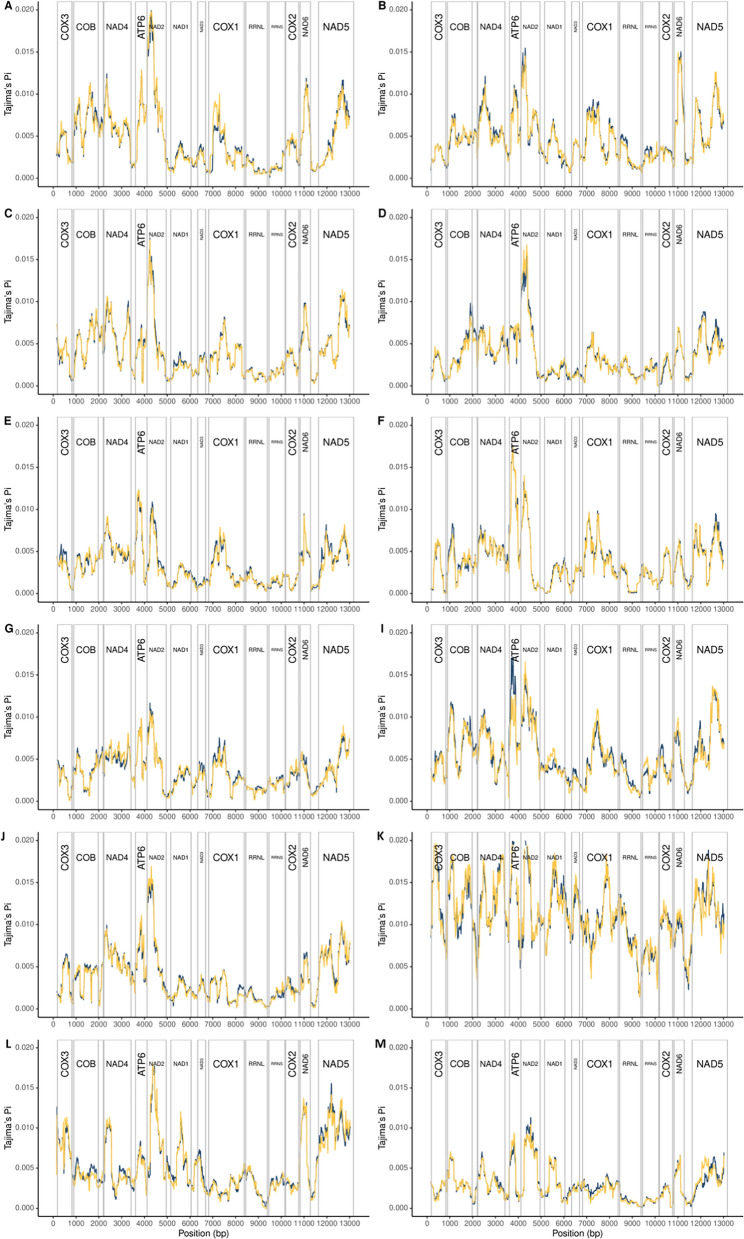
Sliding window analyses (window size 300 bp, step size 10 bp) of PoolSeq data to infer nucleotide diversity (Tajima’s π) across the mitogenome of *Kapentagyrus tanganicanus* (pools **A**–**J**) and *Kapentagyrus limnotrissae* (pools **K**–**M**) of coverage 5×/individual (blue line) and 10×/individual (yellow line). Gene boundaries with the respective position in the mitogenome are displayed above the graph. Details on the respective parasite populations are presented in Table 1

The PCGs with the highest nucleotide diversity (Tajima’s π) were NADH dehydrogenase subunits 2 (*nad2*), 6 (*nad6*) and 5 (*nad5*) in most populations of *K. tanganicanus* (Fig. 3A–J) and *K. limnotrissae* from Mpulungu (southern subbasin) sampled in the dry season in September 2018 (Fig. 3L). In the populations of *K. tanganicanus* ex *L. miodon* from Kalemie (central subbasin), *atp6* showed somewhat elevated levels of variation compared to the other PCGs (Fig. 3E, F). A similar pattern was observed in the population of *K. tanganicanus* ex *S. tanganicae* from Mpulungu (southern subbasin) (Fig. 3I). In the case of *K. limnotrissae*, contrasting patterns of variation across the mitogenome were found in the rainy season with elevated levels across all PCGs in the population from Mpulungu (April 2018) and decreased variation in the population from Kalemie collected in April 2018 (Fig. 3K–M).

Overall negative values of Tajima’s D across all PCGs were detected with no clear pattern related to the number of pooled individuals, subbasin origin or the identity of the host-species across the subsampled coverage/individual between species of *Kapentagyrus* and across populations of *K. tanganicanus* (see Table S2, Fig. S3).

**Fig. 4:**
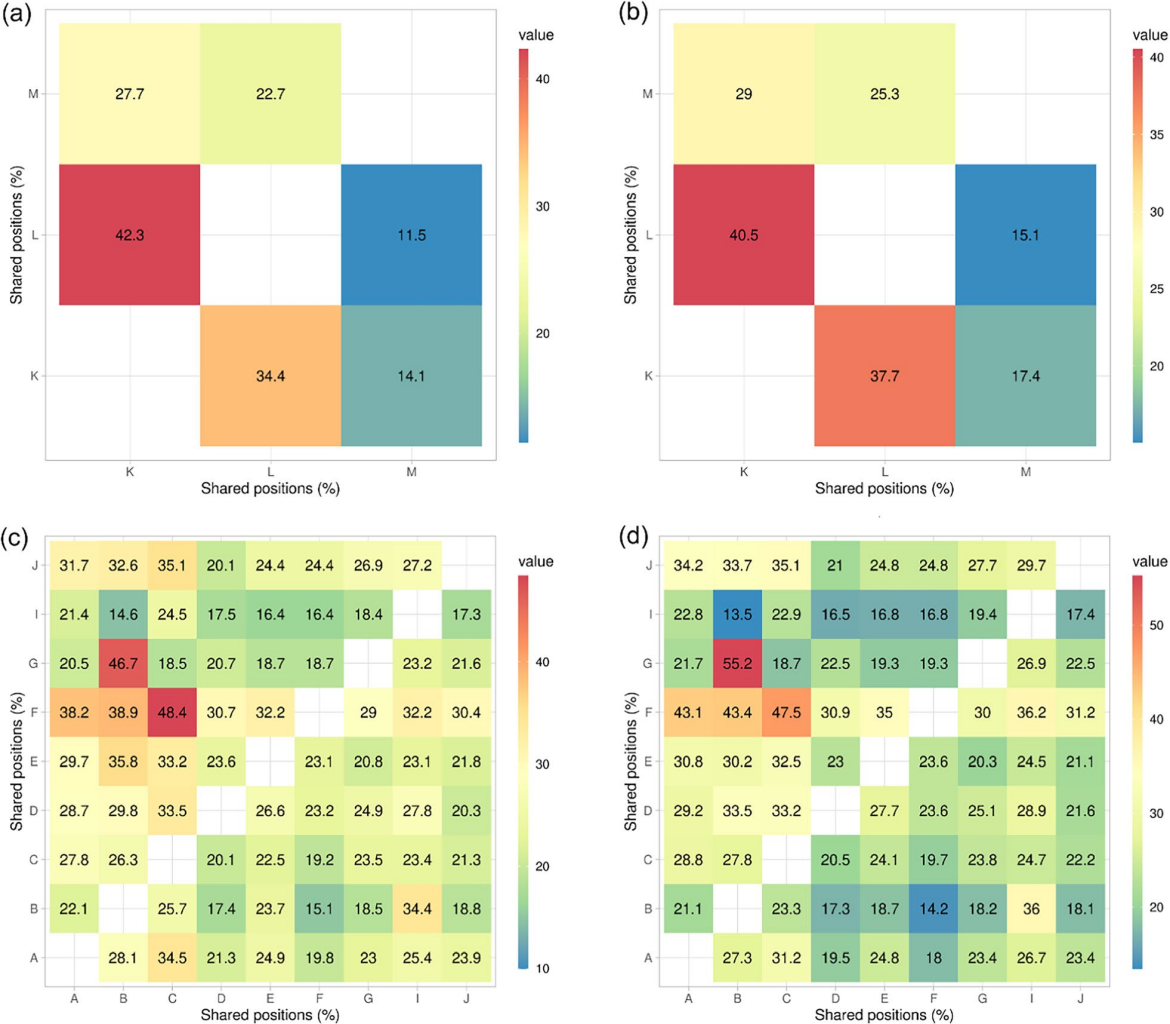
Heatmaps comparing number of shared SNP positions (%) across the mitogenome (noncoding regions located between *nad5* and *trnG* gene regions were excluded) between the pools of coverage 5×/individual (**a**, **c**) and 10×/individual (**b**, **d**) of *K. limnotrissae* (pools K–M) and *K. tanganicanus* (A–J). The percentage is proportional to the total number of SNP positions in the respective pool below and above diagonal. Details on the respective parasite populations are presented in Table 1

The relatively low number of shared SNP positions (between 13.5 and 55.2%) among the analysed populations indicated that the variation detected was to a large extent population-specific (see Fig. 4). The application of different coverage filters (5×/individual vs. 10×/individual) didn’t have a strong influence on the results.

**Fig. 5:**
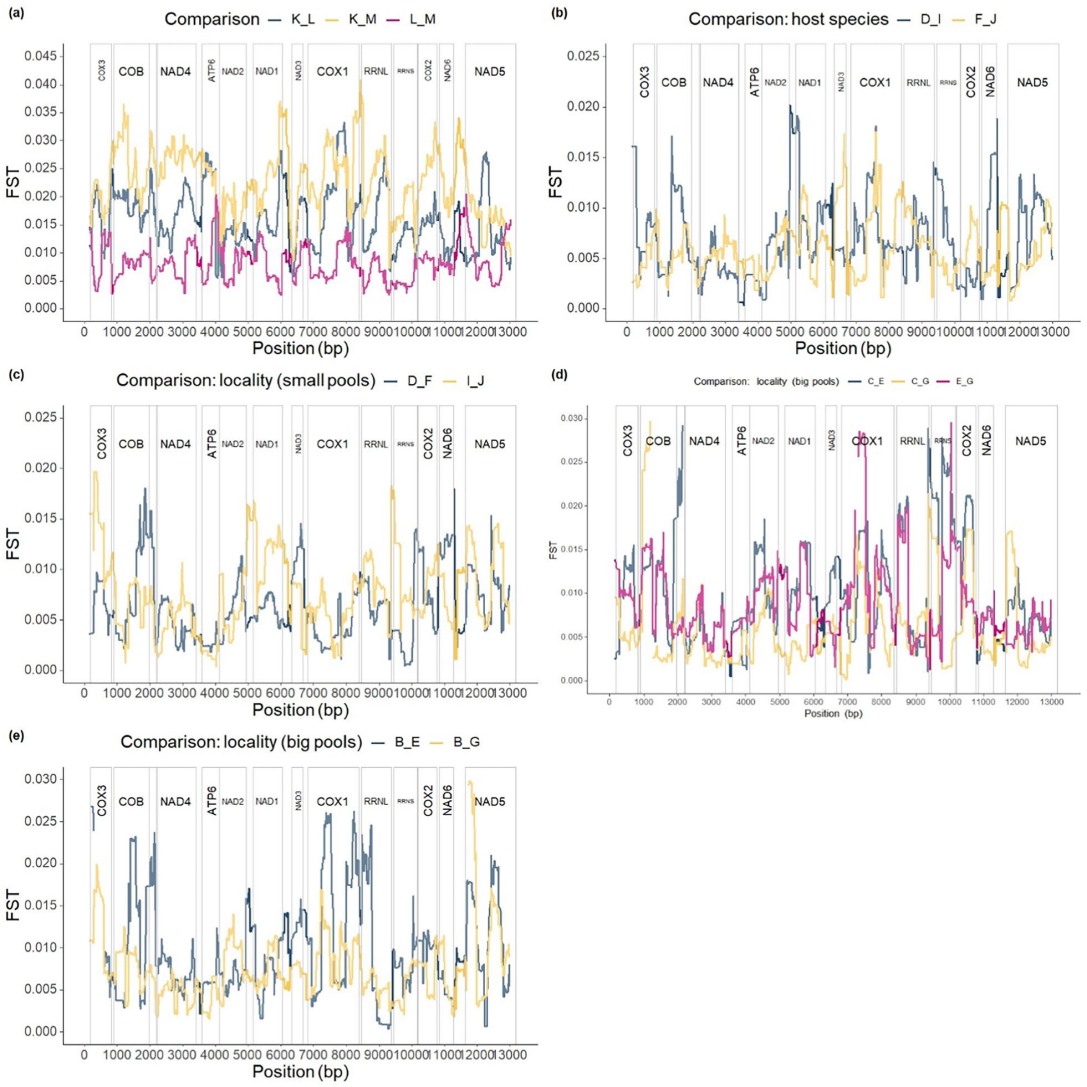
Pairwise comparison of FST values between populations of *K. limnotrissae* (**a**) and *K. tanganicanus* (**b**–**e**), respectively. Details on the respective parasite populations are presented in Table 1

**Fig. 6:**
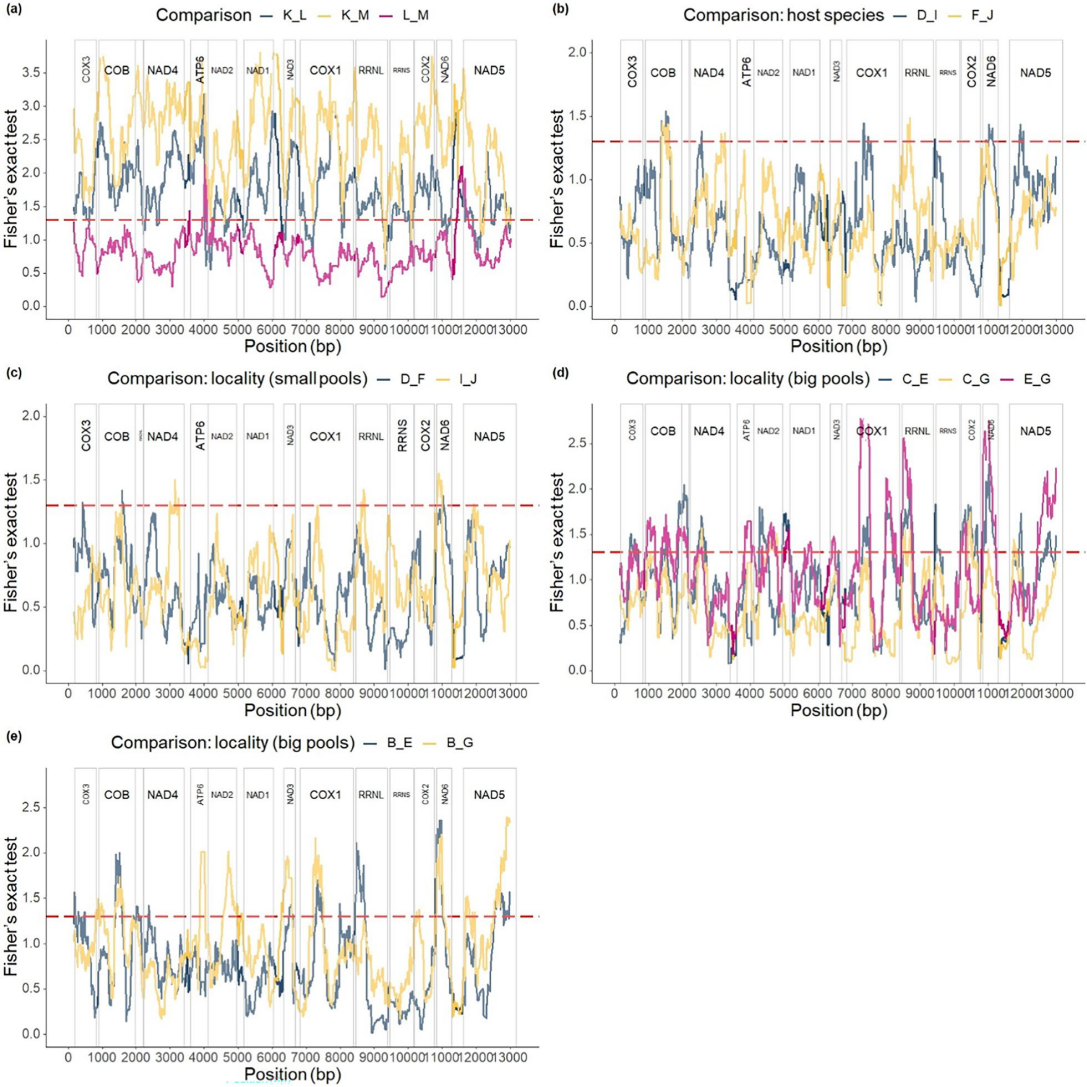
Pairwise comparisons of Fisher’s exact test values between the populations of *K. limnotrissae* (**a**) and *K. tanganicanus* (**b**–**e**), respectively. The dotted red line refers to the p-value of 0.05. Details on the respective parasite populations are presented in Table 1

**Fig. 7:**
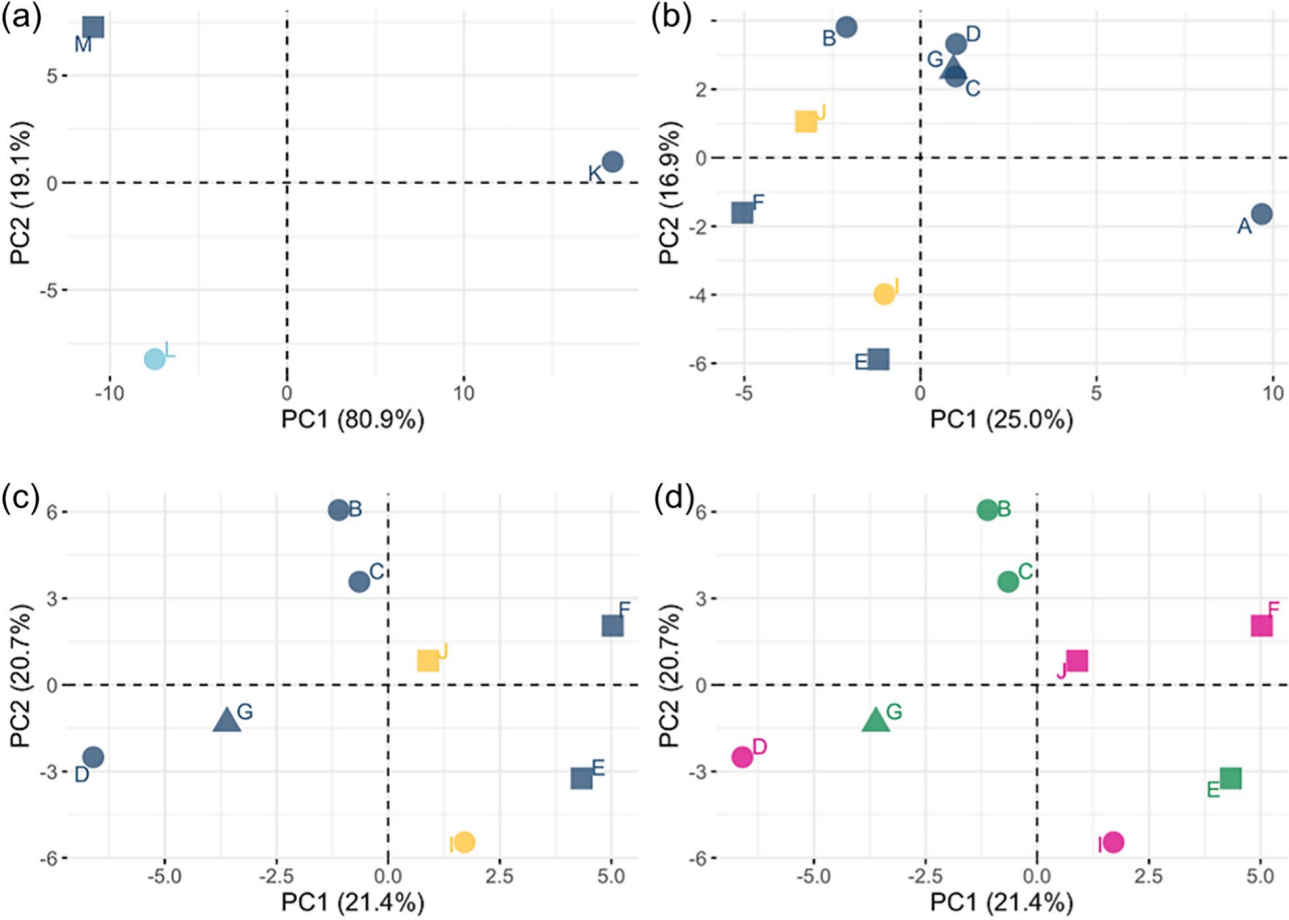
Principal component analysis (the first two axes are plotted) based on frequencies of major shared SNP variants in the mitochondrial genome among populations of *K. limnotrissae* (**a**), all populations of *K. tanganicanus* (**b**) and populations of *K. tanganicanus* except pool A as an outlier (**c**, **d**). Localities—Kalemie (square), Mpulungu (circle), Nsumbu Bay (triangle). Host species—*L. miodon* (blue), *S. tanganicae* (yellow). Sampling months—April (blue), September (light blue). Pool sizes: n = 80–90 (green), n = 56–60 (pink). Details on the respective parasite populations are presented in Table 1

**Fig. 8:**
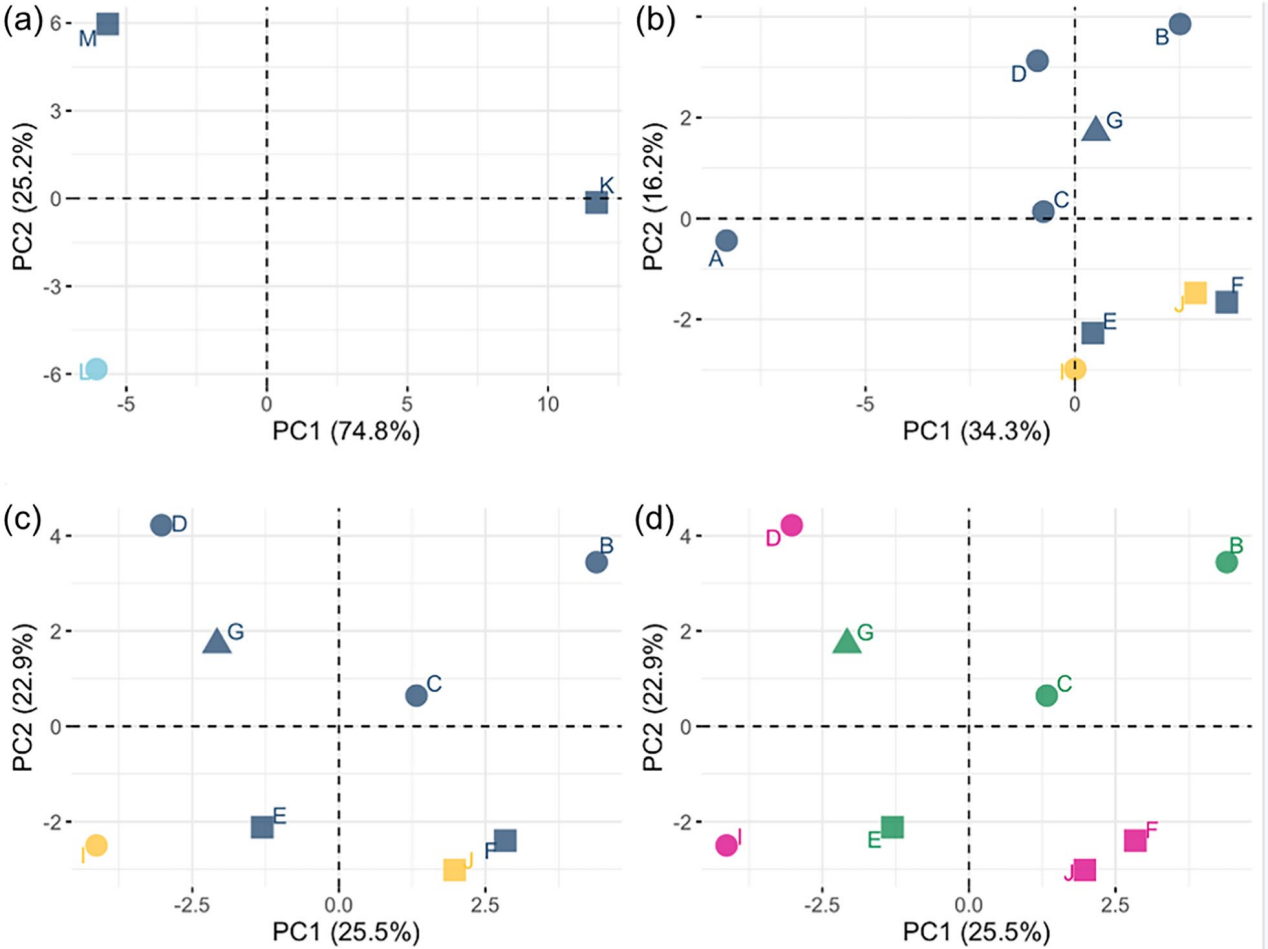
Principal component analysis based on frequencies of major shared SNP variants applying min. frequency of the minor SNPs variant to 0.01 in the mitochondrial genome among populations of *K. limnotrissae* (panel **a**) all populations of *K. tanganicanus* (panel **b**) and populations of *K. tanganicanus* except pool A as an outlier (**c**, **d**). Localities—Kalemie (square), Mpulungu (circle), Nsumbu Bay (triangle). Host species—*L. miodon* (blue), *S. tanganicae* (yellow). Sampling months—April (blue), September (light blue). Pool sizes—n = 80–90 (green), n = 56–60 (pink). Details on the respective parasite populations are presented in Table 1

In *K. limnotrissae*, significant differentiation of the SNP variant frequencies across the mitogenome between some of the populations was observed (Fig. 5a and Table 2) with similar levels of differentiation across PCGs (Figs. 5, 6a). The PCAs based on the major SNP variant showed differentiation among the populations of *K. limnotrissae* along both PC axes (Fig. 7a), also when applying a min. frequency of the SNP variant of 0.01 (Fig. 8a) and 0.05 (Fig. S4a in Additional file 1).

**Table 2:** Pairwise comparisons of population differentiation between the populations of *K. limnotrissae* and *K. tanganicanus* of coverage 5x/individual and min. number of the SNP variant of 2

*K. limnotrissae*			
	K	L	M
K	x	0.01606	0.02306
L	**1.72415**	x	0.00828
M	**2.53691**	0.83443	x

*K. tanganicanus*								
	B	C	D	E	F	G	I	J
B	x	0.00729	0.00672	0.00779	0.00942	0.00682	0.00617	0.00770
C	0.57706	x	0.00878	0.01116	0.00743	0.00852	0.00673	0.00656
D	0.47760	0.55771	x	0.00723	0.00995	0.00692	0.00741	0.00928
E	0.53939	0.66538	0.52203	x	0.01264	0.00680	0.00960	0.01234
F	0.43076	0.58572	0.44468	0.52372	x	0.00905	0.00874	0.00774
G	0.66160	0.51145	0.63323	0.76811	0.67804	x	0.00791	0.00878
I	0.47764	0.59407	0.44401	0.52446	0.42854	0.65764	x	0.00789
J	0.43330	0.58031	0.42285	0.49318	0.42314	0.66052	0.43323	x

Values of Fisher’s exact test and the respective −log10(*p*-value) with comparisons below the *p*-value of 0.05 highlighted in bold (below the diagonal) and FST values (above the diagonal)

Geographic and seasonal population structure analyses revealed no significant differences in any of the pairwise intraspecific comparisons across the entire mitogenome in *K. tanganicanus* (Figs. 5, 6b–e, Table 2). There was also no population structuring of *K. tanganicanus* related to host species. In *K. tanganicanus*, however, pool A (metapopulation of 80 individuals from Mpulungu in April 2018 that was intentionally sequenced with lower coverage) was separated along PC2 also when applying a min. frequency of the rare SNP variant of 0.01 (Fig. 7b) and 0.05 (Fig. S4b). However, pool A comprised a substantially lower number of reads and was considered an outlier in the analyses. After omitting pool A, there was no clear separation of populations related to locality of origin, pool size or host species in any of the PCAs of *K. tanganicanus* (Figs. 7b–d, 8b–d, S2b–d).

A comparison with previously published results on a portion of the *cox1* gene using Sanger sequencing of individual parasites [41] revealed a similar level of nucleotide diversity (Tajima’s π), Tajima’s D and number of SNPs in both parasite species (see Table 3 for more details).

**Table 3:** Population-level nucleotide diversity and Tajima’s D of species of *Kapentagyrus* inferred from part (415 bp) of the *cox1* gene based on PoolSeq (coverage 5× and 10x/individual, min. number of SNP variant = 2) and on Sanger data [41], respectively

Pool ID	Population size		Locality	Subbasin	Date	Population	Host species	Coverage/individual	Tajima’s π	Tajima’s D	SNPs
*Kapentagyrus tanganicanus*											
A	80		Mpulungu	South	12.04.2018	Metapopulation	*L. miodon*	5×	0.00193	−2.74392	10
								10×	0.00201	−3.01454	14
B	85		Mpulungu	South	12.04.2018	Metapopulation	*L. miodon*	5×	0.00327	−3.01113	17
								10×	0.00368	−3.00644	19
C	90		Mpulungu	South	19.04.2108	Infrapopulation	*L. miodon*	5×	0.00380	−2.77835	19
								10×	0.00354	−2.83547	19
D	60		Mpulungu	South	19.04.2108	Metapopulation	*L. miodon*	5×	0.00318	−2.53654	11
								10×	0.00295	−2.61601	11
E	90		Kalemie	Central	12.04.2018	Metapopulation	*L. miodon*	5×	0.00259	−2.65879	9
								10×	0.00274	−2.80902	11
F	60		Kalemie	Central	12.04.2018	Metapopulation	*L. miodon*	5×	0.00501	−2.20116	10
								10×	0.00487	−2.20347	10
G	90		Nsumbu Bay	South	Apr.18	Metapopulation	*L. miodon*	5×	0.00213	−2.83455	9
								10×	0.00236	−2.61169	8
I	56		Mpulungu	South	Apr.18	Metapopulation	*S. tanganicae*	5×	0.00570	−2.98751	22
								10×	0.00508	−3.27550	29
J	59		Kalemie	Central	12.04.2018	Metapopulation	*S. tanganicae*	5×	0.00108	−2.21300	3
								10×	0.00087	−2.39072	3
*Kapentagyrus limnotrissae*											
K		59	Mpulungu	South	Apr.18	Metapopulation	*L. miodon*	5×	0.01386	−1.72211	21
								10×	0.01379	−1.71416	21
L		58	Mpulungu	South	Sept. 18	Metapopulation	*L. miodon*	5×	0.00233	−3.32629	11
								10×	0.00235	−3.41089	13
M		59	Kalemie	Central	12.04.2018	Metapopulation	*L. miodon*	5×	0.00233	−2.96402	12
								10×	0.00267	−3.22417	19

No. of individuals (Sanger sequencing)	Locality	Subbasin	Date	Population	Host species	Tajima’s π	Tajima’s D	Snps
*Kapentagyrus tanganicanus*								
195	Baraka, Kalemie, Mpulungu, Uvira	All	April 2016, 2017, 2018	Suprapopulation	*L. miodon* and *S. tanganicae*	0.00380	−2.41	68
21	Kalemie	Central	12.08.2016	Metapopulation	*L. miodon*	0.00314	−1.37385	8
35	Mpulungu	South	12.04.2018	Metapopulation	*L. miodon* and *S. tanganicae*	0.00337	−2.41086	20
39	Uvira	North	12.04.2018	Metapopulation	*L. miodon*	0.00280	−1.82930	12
68	Uvira	North	12.04.2018	Metapopulation	*L. miodon* and *S. tanganicae*	0.00230	−2.17756	17
*Kapentagyrus limnotrissae*								
51	Kalemie, Mpulungu, Uvira	All	April 2016, 2018	Metapopulation	*L. miodon*	0.00224	−2.48	18
23	Kalemie	Central	12.04.2018	Metapopulation	*L. miodon*	0.00267	−1.99456	10

## Discussion

### Population genetic structure of monopisthocotylans is consistent with migration patterns of their pelagic hosts

Mitogenomes of the two endemic monopisthocotylan flatworms *K. tanganicanus* and *K. limnotrissae* infecting endemic pelagic dorosomatid fishes in Lake Tanganyika were PoolSeq-ed, assembled and annotated. To overcome potential shortcomings of using only short *cox1* sequences, we analysed the population structure across all mitochondrial PCGs over a geographic and seasonal gradient in both targeted monopisthocotylan parasites. Unlike the results of the previous single gene study by Kmentová et al*.* [41], the results of the current study concurred with the previously reported life-style differences (dispersal restrictions during the littoral phase of *L. miodon*) between the two dorosomatid fishes in Lake Tanganyika [17, 74, 75] and their shallow geographical population structure [13, 15]. This is visible in the pattern of restricted gene flow of *K. limnotrissae* reported in the current study, a parasite species infecting only a littoral and residental stage of *L. miodon* [15]. Strikingly, our study targeting parasites revealed the same geographic pattern from the parasites’ mitogenomes that Junker et al*.* [15] reported for the dorosomatid hosts based on genome wide RAD-seq data.

Due to their direct life cycle and short generation time, monopisthocotylans have been proposed as potentially ideal tags for population structure of their hosts through magnifying the effects of migration barriers [76–78]. However, only a handful of studies on monopisthocotylan population connectivity have been conducted so far. Despite shared ecological and host-range characteristics, contrasting phylogeographic structure of monopisthocotylan parasites infecting butterflyfishes (Chaetodontidae) in the South Pacific Ocean has been suggested [79]. Various parameters have been suggested as drivers of contrasting patterns of monopisthocotylan population structure. Population structuring of *Zeuxapta seriolae* (Meserve, 1938), a parasite of *Seriola lalandi*, a benthopelagic fish species with a circumpolar distribution, is enhanced by environmental differences reflected in contrasting egg production levels between studied localities [20]. Additionally, it has been hypothesized that the evolutionary age of the parasite species, dispersal capabilities of egg and larval stages [79] and host specificity [80, 81] are potentially important factors determining population structuring in monopisthocotylan parasites. The differences in gene flow between the populations of the studied parasite species revealed by the increased resolution of whole PCG data can be explained by *K. limnotrissae* being strictly host-specific to *L. miodon*. Conversely, the lack of geographical structure in *K. tanganicanus* may be a direct consequence of its broader host range, as long-distance migration and lack of spawning ground fidelity were reported for its presumably most mobile host *S. tanganicae* [12, 15]. The lack of population structure observed in *K. tanganicanus* may be therefore explained by the lake’s currents and the species’ association with pelagic hosts, which likely facilitate long-distance dispersal of eggs and other life stages, thereby preventing population structuring or isolation along the North–South axis. These results further advocate for harmonization of fisheries management across the lake’s subbasins to ensure effective management of this important protein source at the international level [82]. Given the profound seasonally driven fluctuations in dorosomatid abundance along the North–South axis of Lake Tanganyika [17, 83] and the current sampling lacking temporal coverage, conclusions related to drivers of the reported patterns will further need to be verified across seasons and employing a larger number of genomic pools across all three host-parasite combinations.

Our results are in line with the hypothesis of parasites mirroring population structure of the most mobile hosts when parasites infect multiple hosts [84, 85]. A similar phenomenon has been suggested for *Cichlidogyrus casuarinus* Pariselle, Muterezi Bukinga & Vanhove, 2015, a monopisthocotylan species infecting pelagic cichlid species of Bathybatini in Lake Tanganyika [43] and for species of *Cichlidogyru*s infecting tilapia in native and introduced areas [81]. As it is notoriously difficult to generate high-quality genomic data from minute monopisthocotylan flatworms of wildlife origin, a PoolSeq methodology was employed to obtain mitochondrial genomes. However, future studies targeting whole genome level differentiation are needed to further understand gene flow dynamics and potential North–South differentiation in these parasitic flatworms in Lake Tanganyika.

### Contrasting patterns of mitochondrial gene variation in monopisthocotylan flatworms

The *cox1* fragment that is usually applied as an informative marker for population studies in parasitic flatworms [20, 86–88] appeared to be one of the least variable PCGs in both species of *Kapentagyrus*. This observation may also largely explain why Kmentová et al*.* [41] did not find any geographic population structure in *K. limnotrissae*. Thus, this study supported the suggestion that other mitochondrial genes than the *cox1* gene should be used to assess population structure and especially recent gene flow barriers for parasitic flatworms (e.g., Zhang et al*.* [89]). More informative markers may be some of the PCGs from either the dehydrogenase gene family or *atp6*, as also previously suggested for other lineages of monopisthocotylans such as *Cichlidogyrus* [43, 81] or marine diplectanids [89].

The phylogenetic position of *Kapentagyrus* is still unclear, as no apparent sister-group has been identified do far [90], but given the relatively recent intralacustrine diversification of dorosomatids in the lake [91, 92], diversification between the two Lake Tanganyika species of *Kapentagyrus* has been suggested to be more recent than that of *C. casuarinus*, a monopisthocotylan species infecting pelagic cichlid hosts and of which lake-wide population structure and mitogenome level diversity have been previously studied [43, 93]. While the overall level of nucleotide diversity was similar, the pattern of relative variation among mitochondrial PCGs showed larger differences between the mitogenome of *C. casuarinus* and *Kapentagyrus* spp. In the present study, the overall similarity in the level of mitochondrial nucleotide diversity between the two studied monopisthocotylan species suggested similar effective population sizes. Consistent with the results of the present study, negative values of Tajima’s D that may suggest purifying selection in mitochondrial genomes of flatworms have been observed in previous studies [43, 94, 95]. Changes of evolutionary forces across the PCGs over time was suggested [96]. The impressive phenotypic diversity and species richness of parasitic flatworms are reflected in their vast range of life strategies, which is likely correlated with their genomic variation [97, 98]. Previously, mutation rather than natural selection was suggested as cause of observed high mitochondrial genetic diversity with no signs of relaxed selection in parasitic flatworms [99]. Given the contrasting results, future studies are needed to close the knowledge gap on the evolutionary mechanisms linking microevolution and macroevolution of parasitic flatworms.

### The role of population size

The relatively large number of singletons, low number of shared SNPs between populations and negative Tajima’s D values across the PCGs may not only be explained by purifying selection as argued above, but could also reflect expanding panmictic populations of both parasite species [100, 101].

Indeed, fluctuations in population size are profound in both studied monopisthocotylan species due to their overdispersion [23]. Since lake-level fluctuations are expected to have only little consequences for pelagic organisms compared to littoral ones [102, 103], the seasonal migrations and short generation times of dorosomatids in the lake might be the main factors determining their effective population size and diversification potential [12, 13, 15] and subsequently those of their monopisthocotylan parasites. In the previously studied *cox1* region, both studied monopisthocotylan species displayed one major haplotype, and a high proportion of singletons pointed at recent population expansion and/or fluctuations [41]. Results of neutrality tests on the *cox1* region [41] indicated recent population expansion for both studied monopisthocotylan species. Their populations were suggested to be homogenised by lake-wide migration. Moreover, the lack of seasonal diversification in the parasite mitogenome sequences did not provide evidence for strong environmentally driven selection within the temporal window sampled.

### PoolSeq as a sequencing strategy for field-retrieved flatworm populations

The current study applied a PoolSeq strategy [48–50] to assess population level diversification of field-retrieved populations of monopisthocotylans to overcome the challenges of typically low DNA yield per individual. The comparison with the individual-based sequencing using the *cox1* gene portion confirmed the suitability of PoolSeq (see Table 3). Furthermore, no correlation between pool size and the level of diversity was found, suggesting that a number of pooled individuals <60 is sufficient to correctly infer intra-population variation. However, the two approaches differed in the estimated level of population differentiation in relation to subsampled coverage and minimum count of SNP variants. Therefore, uniform coverage and minimum count of SNP variants over different studied populations, standardized DNA input per individual where possible and validation of key patterns with individual-based sequencing for a subset of populations/markers as well as caution towards the conclusions about metapopulation structure are recommended to be applied in future mitochondrial-level PoolSeq studies on hardly accessible and low input non model taxa. Finally, the observed patterns should be verified using a whole genome approach to ensure full complementarity with the approach applied on the fish hosts.

## Supplementary Information

Additional file1 (DOCX 1815 kb)

## Data Availability

Raw Illumina reads were submitted to SRA (accession numbers: SRR22937639-50) under BioProject accession PRJNA916857. Parasite specimens were deposited in the collection of the Research Group Zoology: Biodiversity & Toxicology at Hasselt University (Diepenbeek, Belgium) under accession numbers HU.XX.1.01-HU.XX.4.50, HU.XXI.1.01-HU.XXI.4.50 and HU.XXII.1.01-HU.XXII.2.50. Fieldwork was carried out with the approval of the competent local authorities under mission statement 002/MINRST/CRH-U/2018 and the permission of the Fisheries Department of Zambia and under a study permit issued by the government of Zambia (SP 008732).
