# Supplementary material for: Geographic differentiation of ectoparasitic flatworms in the pelagic zone of Lake Tanganyika, Africa
Source: Front Zool. 2026 May 11;23:20. doi: 10.1186/s12983-026-00602-9 (PMC13159304; doi:10.1186/s12983-026-00602-9)
Supplement: Supplementary file 1 — Additional file1 (DOCX 1815 kb) [file 12983_2026_602_MOESM1_ESM.docx]

**Supplementary figures**


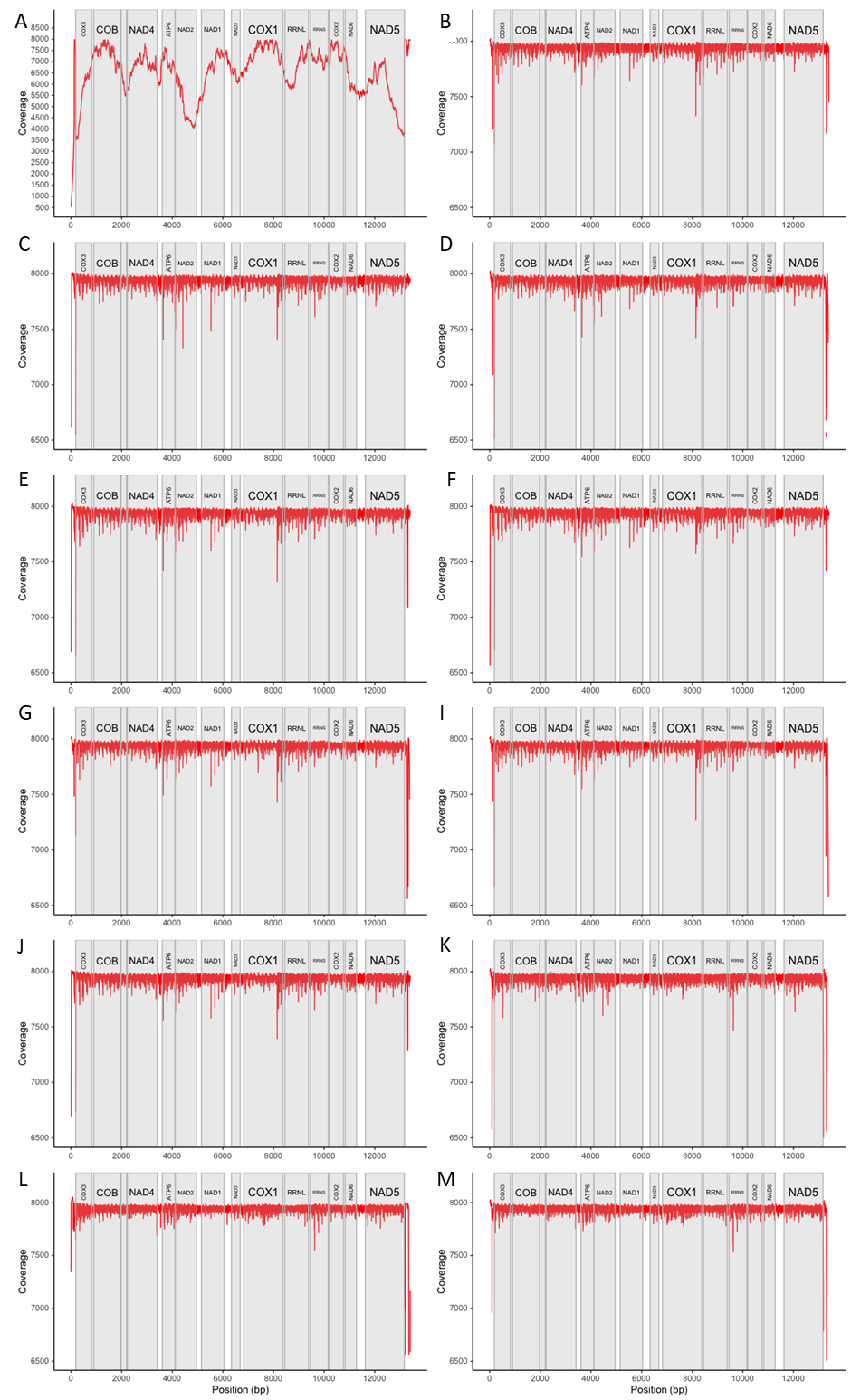


**Fig. S1:** Coverage across mitogenome per pool of *Kapentagyrus tanganicanus* (panels A-I) and *Kapentagyrus limnotrissae* (panels K-L). Details on the respective parasite populations are presented in Table 1 of the main manuscript file.


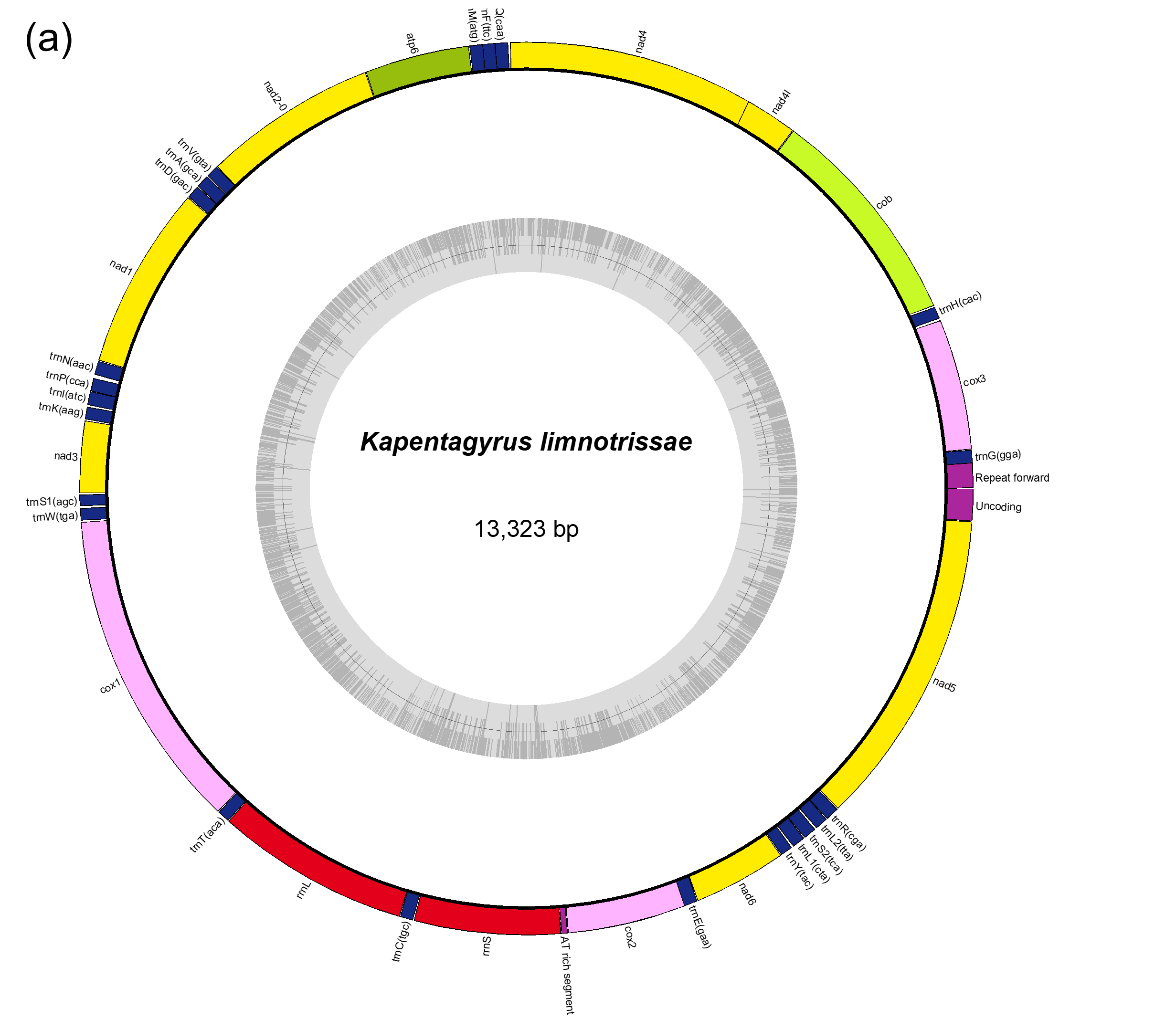


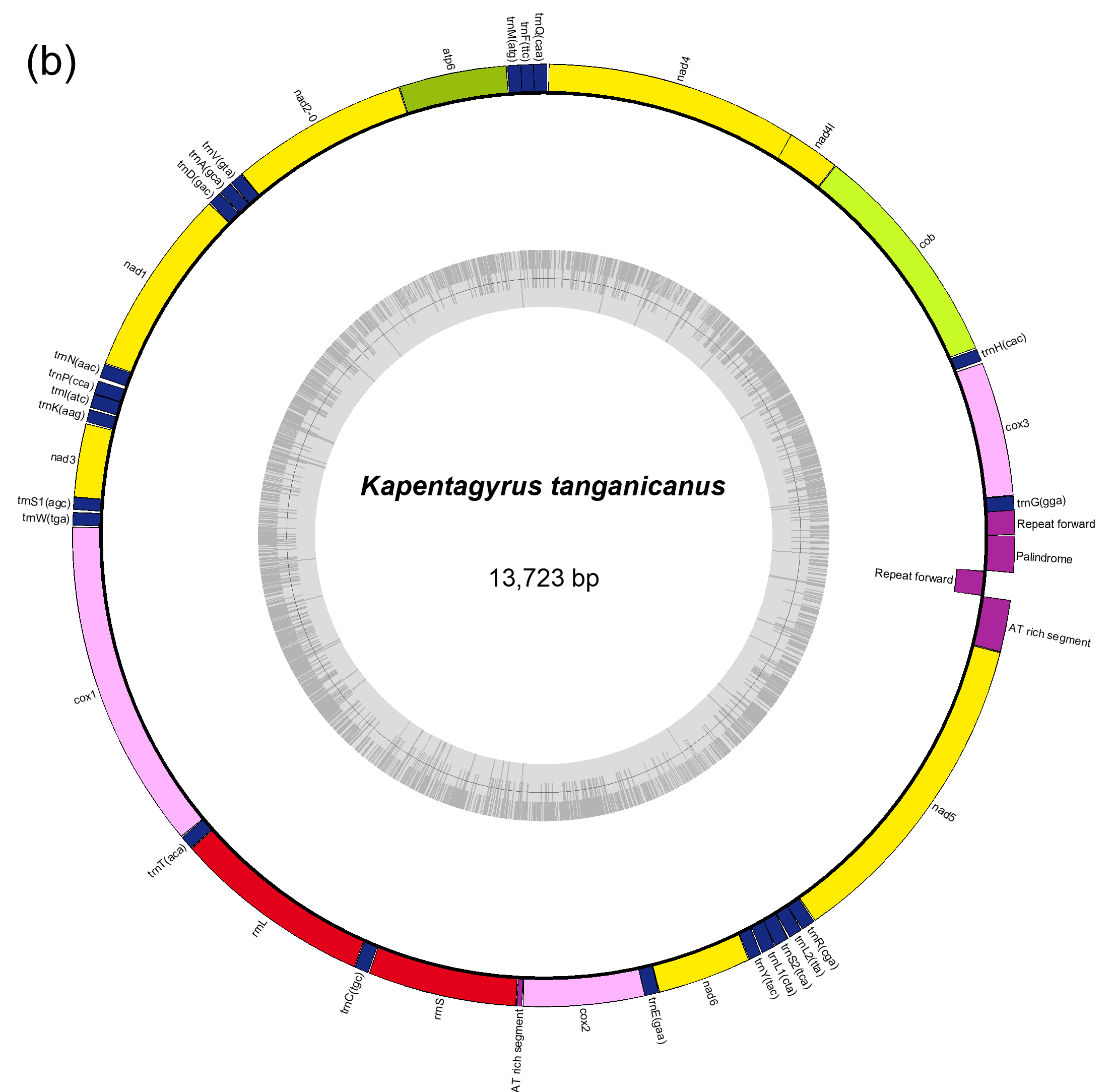


**Fig. S2**: Mitochondrial genomes of *Kapentagyrus tanganicanus* (a) and *Kapentagyrus limnotrissae* (b) with GC content (inner circle).


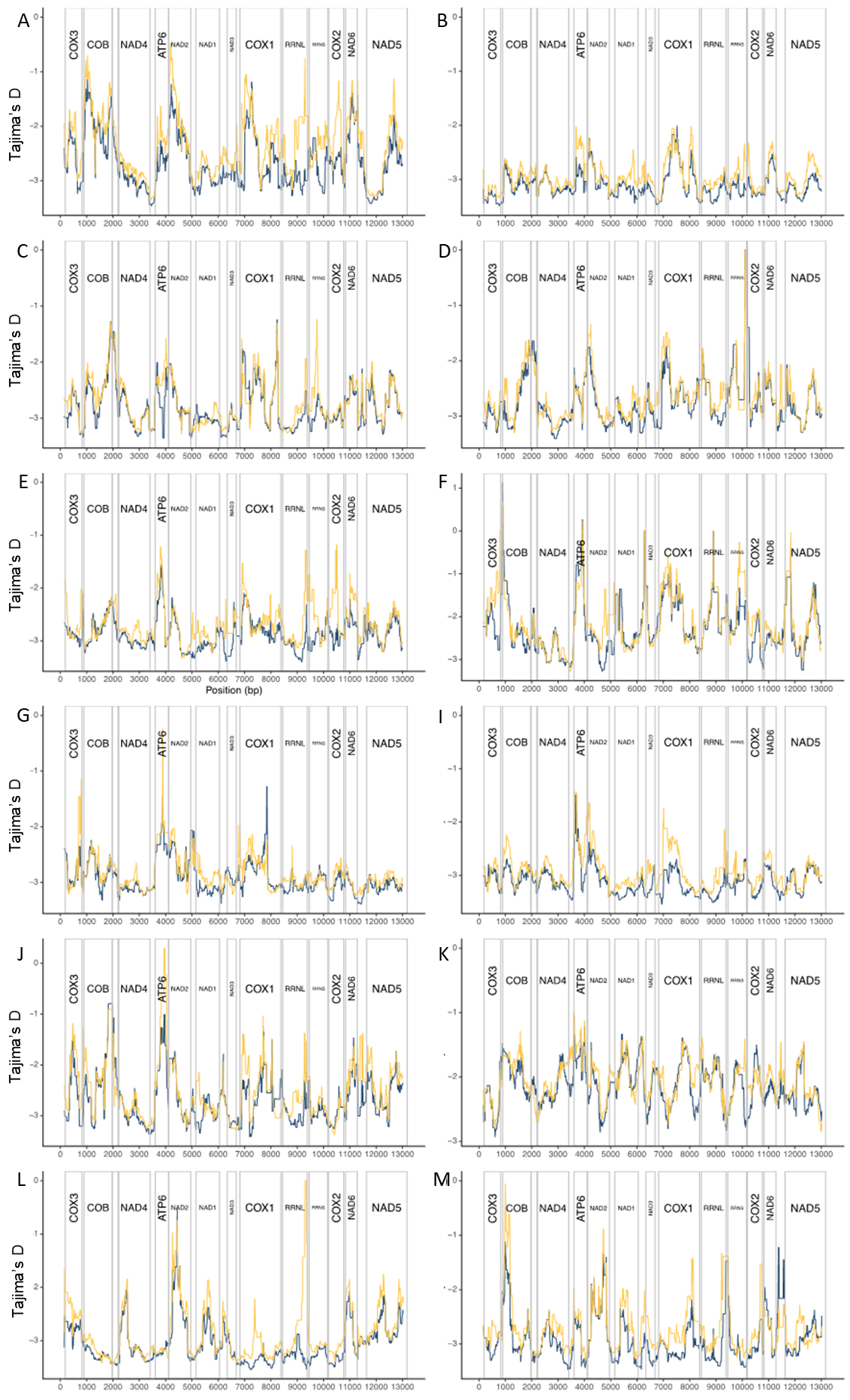


**Fig. S3**: Sliding window analyses (window size 300 bp, step size 10 bp) of PoolSeq data to infer Tajima’s D across the mitogenome of *Kapentagyrus tanganicanus* (pools A - J) and *Kapentagyrus limnotrissae* (pools K - M) of coverage 5x/individual (blue line) and 10x/individual (yellow line). Gene boundaries with the respective position in the mitogenome are displayed above the graph. Details on the respective parasite populations are presented in Table 1 of the main manuscript file.


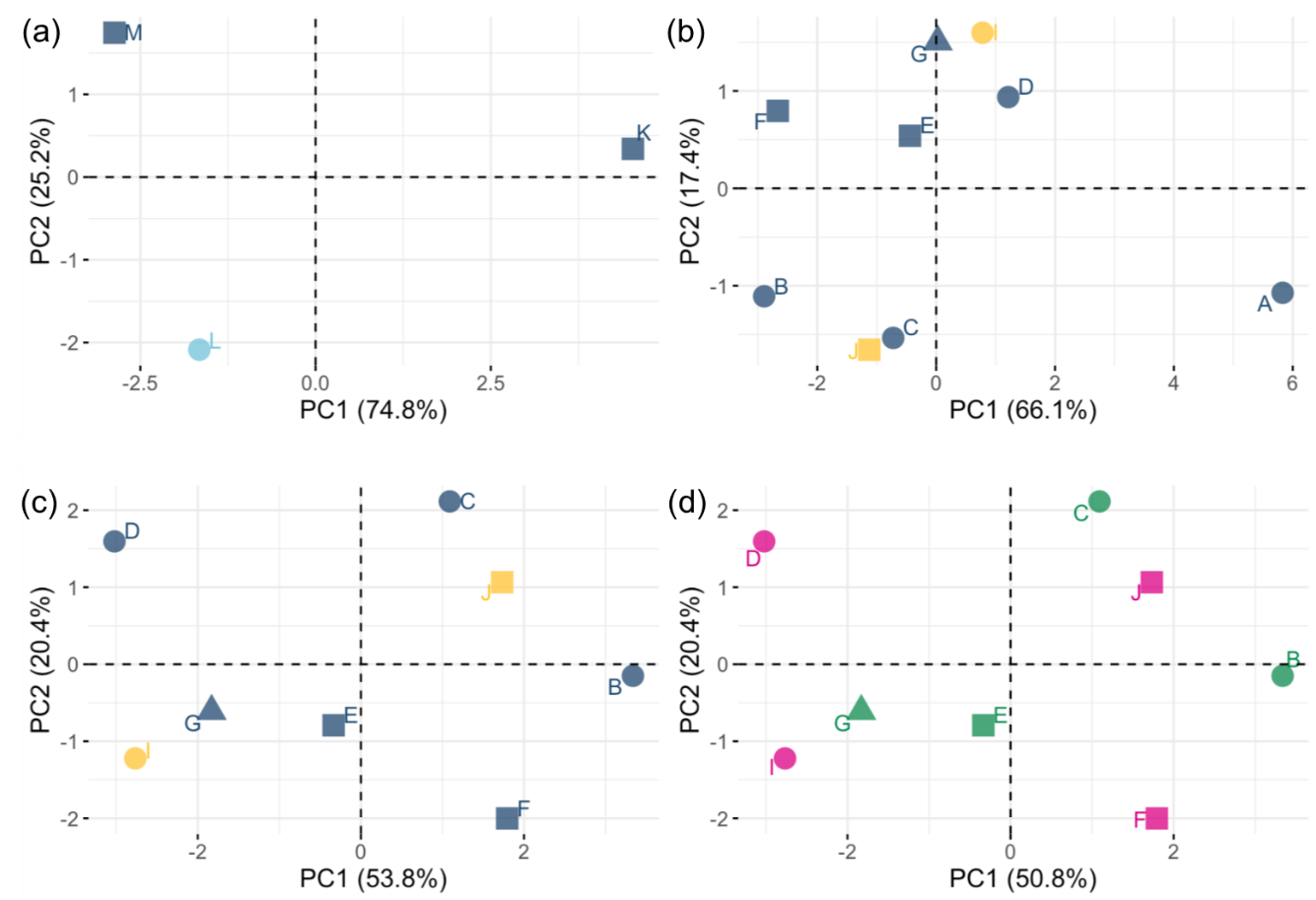


**Fig. S4**: Principal component analyses based on major frequencies of shared SNP variants applying min. frequency of the rare SNP variant to 0.05 in the mitochondrial genome between populations of *K. limnotrissae* (panel A) all populations of *K. tanganicanus* (panel B) and populations of *K. tanganicanus* except pool A as an outlier (C, D). Localities - Kalemie (square), Mpulungu (circle), Nsumbu Bay (triangle). Host species - *L. modon* (blue), *S. tanganicase* (yellow). Sampling months - April (blue), August (light blue). Pool sizes - n= 80 – 90 (green), n= 56 – 60 (pink). Details on the respective parasite populations are presented in Table 1 of the main manuscript file.

**Supplementary tables**

**Table S1**: Overview of the length (in bp) of mitochondrial regions, the start and stop codons (protein-coding genes) and anticodons (tRNA genes) in the assembled mitogenomes of *Kapentagyrus limnotrissae* and *Kapentagyrus tanganicanus*, respectively, being consistent across all analysed populations.

|  | *Kapentagyrus limnotrissae* | | | *Kapentagyrus tanganicanus* | | |
| --- | --- | --- | --- | --- | --- | --- |
| Region | **Length** | **Start/stop codon** | **Anticodon** | **Length** | **Start/stop codon** | **Anticodon** |
| trnG | 67 |  | TCC | 71 |  | TCC |
| cox3 | 638 | ATG/TA |  | 645 | ATG/T |  |
| trnH | 62 |  | GTG | 62 |  | GTG |
| cytb | 1,076 | ATG/TA |  | 1,076 | ATG/TA |  |
| nad4L | 249 | ATG/TAA |  | 249 | ATG/TAA |  |
| nad4 | 1,215 | ATG/TAA |  | 1,215 | ATG/TAA |  |
| trnQ | 65 |  | TTG | 68 |  | TTG |
| trnF | 65 |  | GAA | 65 |  | GAA |
| trnM | 70 |  | CAT | 70 |  | CAT |
| atp6 | 509 | ATG/TA |  | 509 | ATG/TA |  |
| nad2 | 840 | ATG/TAG |  | 840 | ATG/TAG |  |
| trnV | 64 |  | TAC | 65 |  | TAC |
| trnA | 67 |  | TGC | 67 |  | TGC |
| trnD | 67 |  | GTC | 67 |  | GTC |
| nad1 | 891 | ATG/TAA |  | 891 | ATG/TAA |  |
| trnN | 70 |  | GTT | 70 |  | GTT |
| trnP | 67 |  | TGG | 67 |  | TGG |
| trnI | 69 |  | GAT | 69 |  | GAT |
| trnK | 63 |  | CTT | 64 |  | CTT |
| nad3 | 348 | GTG/TAA |  | 348 | GTG/TAA |  |
| trnS1 | 67 |  | GCT | 62 |  | GCT |
| trnW | 63 |  | TCA | 65 |  | TCA |
| cox1 | 1,557 | ATG/TAA |  | 1,557 | ATG/TAA |  |
| trnT | 64 |  | TGT | 68 |  | TGT |
| 16S rRNA | 940 |  |  | 939 |  |  |
| trnC | 66 |  | GCA | 71 |  | GCA |
| 12S rRNA | 707 |  |  | 706 |  |  |
| AT rich segment | 32 |  | 9.4% GC | 28 |  | 7.1% GC |
| cox2 | 585 | ATG/TAG |  | 558 | ATG/TAG |  |
| trnE | 69 |  | TTC | 72 |  | TTC |
| nad6 | 450 | GTG/TAA |  | 450 | ATG/TAA |  |
| trnY | 63 |  | GTA | 64 |  | GTA |
| trnL1 | 68 |  | TAG | 70 |  | TAG |
| trnS2 | 67 |  | TGA | 73 |  | TGA |
| trnL2 | 67 |  | TAA | 69 |  | TAA |
| trnR | 69 |  | TCG | 69 |  | TCG |
| nad5 | 1,540 | ATG/TAA |  | 1,545 | GTG/TAA |  |
| AT rich segment | 157 |  | 21.8% GC | 247 |  | 13.8% GC |
| Repeated reverse | / |  | / | 125 |  | 12% GC |
| Palindrome | / |  | / | 175 |  | 14.3% GC |
| Repeat forward | 126 |  | 11.9% GC | 125 |  | 12% GC |

**Table S2:** Summary of the mean nucleotide diversity (Tajima’s Pi) across the mitogenome between the species of *Kapentagyrus* and populations of *K. tanganicanus*.

| Parasite species | Population/Factor | Coverage/individual | Tajima's Pi | Tajima's D |
| --- | --- | --- | --- | --- |
| *K. limnotrissae* | All | 10x | 0.00636 | -2.78228 |
| *K. tanganicanus* | All | 10x | 0.00396 | -2.89357 |
| *K. limnotrissae* | All | 5x | 0.00632 | -2.61869 |
| *K. tanganicanus* | All | 5x | 0.00388 | -2.76764 |
| *K. tanganicanus* | Central | 10x | 0.00337 | -2.85390 |
| *K. tanganicanus* | South | 10x | 0.00515 | -2.87167 |
| *K. tanganicanus* | Central | 5x | 0.00329 | -2.73092 |
| *K. tanganicanus* | South | 5x | 0.00510 | -2.73015 |
| *K. tanganicanus* | >60 individuals | 10x | 0.00393 | -2.94637 |
| *K. tanganicanus* | <60 individuals | 10x | 0.00501 | -2.80816 |
| *K. tanganicanus* | >60 individuals | 5x | 0.00387 | -2.81172 |
| *K. tanganicanus* | <60 individuals | 5x | 0.00494 | -2.67232 |
| *K. tanganicanus* | *L. miodon* | 10x | 0.00450 | -2.70411 |
| *K. tanganicanus* | *S. tanganicae* | 10x | 0.00445 | -2.86191 |
| *K. tanganicanus* | *L. miodon* | 5x | 0.00456 | -2.83997 |
| *K. tanganicanus* | *S. tanganicae* | 5x | 0.00454 | -2.99464 |
